# Engineering redox homeostasis to develop efficient alcohol-producing microbial cell factories

**DOI:** 10.1186/s12934-017-0728-3

**Published:** 2017-06-24

**Authors:** Chunhua Zhao, Qiuwei Zhao, Yin Li, Yanping Zhang

**Affiliations:** 1 CAS Key Laboratory of Microbial Physiological and Metabolic Engineering, Institute of Microbiology, Chinese Academy of Sciences, No. 1 West Beichen Road Chaoyang District, Beijing, 100101 China; 2 University of Chinese Academy of Sciences, Beijing, 100049 China

**Keywords:** Redox homeostasis, Metabolic engineering, Alcohol, Cofactor engineering, Glutathione, Reducing equivalent

## Abstract

The biosynthetic pathways of most alcohols are linked to intracellular redox homeostasis, which is crucial for life. This crucial balance is primarily controlled by the generation of reducing equivalents, as well as the (reduction)-oxidation metabolic cycle and the thiol redox homeostasis system. As a main oxidation pathway of reducing equivalents, the biosynthesis of most alcohols includes redox reactions, which are dependent on cofactors such as NADH or NADPH. Thus, when engineering alcohol-producing strains, the availability of cofactors and redox homeostasis must be considered. In this review, recent advances on the engineering of cellular redox homeostasis systems to accelerate alcohol biosynthesis are summarized. Recent approaches include improving cofactor availability, manipulating the affinity of redox enzymes to specific cofactors, as well as globally controlling redox reactions, indicating the power of these approaches, and opening a path towards improving the production of a number of different industrially-relevant alcohols in the near future.

## Background

Due to the increasing concerns surrounding limited fossil resources and environmental problems, there has been much interest in the microbial production of chemicals and fuels from renewable resources. Alcohols such as ethanol, 1,3-propanediol, butanol, isobutanol, 2,3-butanediol and 1,4-butanediol, can be used as important platform chemicals or biofuels [1]. Since they are bulk products, the demand for most of these compounds is highly cost-sensitive. To meet this challenge, the microbial cell factories for producing alcohols must be engineered to increase the titer, yield and productivity of target products as much as possible.

Since wild-type microorganisms do not allow the production of industrially relevant alcohols with high enough efficiency, many efforts have been undertaken to improve their production by systems metabolic engineering [2]. To develop microbial strains that maximize the titer, yield and productivity of the target products, intracellular metabolic fluxes must be optimized using various molecular and high-throughput techniques, including, but not limited to: selecting the best biosynthesis genes [3], overexpressing rate-limiting enzymes, fine-tuning the expression of pathway enzymes [4], reinforcing the direct biosynthesis route [5–7], deleting or down-regulating competing pathways [8, 9], as well as deactivating degradation and utilization pathways or removing feedback regulation [10].

Most of the recent successful systems metabolic engineering examples of the development of alcohol-producing microorganisms focused on directly engineering enzymes of the metabolic pathways in question. However, in addition to the activity of enzymes involved in the pathway itself, the metabolic flux also depends on the concentrations of precursors and cofactors in the cells [11]. Since most alcohol production pathways comprise redox reactions, their production efficiency depends on the availability of cofactors. The cofactors in question are usually some type of reducing equivalents, such as NADH and NADPH, which usually act as carriers of electrons generated from substrate oxidation. Under aerobic conditions, the electrons provided by NAD(P)H are commonly ultimately accepted by O_2_ [12], whereby NAD(P)H is converted to its oxidized form. Therefore, since alcohol production is generally performed under anaerobic conditions, the strains maintain their cellular redox balance mainly through the reactions of central metabolism, which are significantly different from aerobic microbial metabolism. By decreasing the amounts of acid-forming enzymes and/or enhancing the butanol synthetic pathway genes expression in the non-sporulating, non-solventogenic *Clostridium acetobutylicum* strain M5, Sillers et al. [13] demonstrated the rigidity of intracellular electron balance. Thus, in order to sustain growth and metabolism, the metabolic network must be tweaked to maintain the redox balance in the cells [14].

Currently, the primary feedstocks used in the biological production of alcohols are sugarcane, sugar beet, maize (corn) and sorghum, due to their low price and wide availability in the market [15]. These feedstocks mainly provide fermentable sugars, which are easily metabolized by the production strains, generating NADH, NADPH, ferredoxin and other reducing equivalents that are needed in the alcohol biosynthetic pathways. However, due to the unfavorable stoichiometry of available electrons from a substrate such as glucose [16], the maximum theoretical yields for alcohols are mostly lower than 0.5 g/g, with the exception of ethanol, at 0.51 g/g [17]. Furthermore, in addition to alcohol synthesis, there are many other pathways that are competing for reducing equivalents, especially in anaerobes, such as hydrogen production [14]. Actually, due to the imbalances between the generation of reducing equivalents from substrates and their oxidation by redox enzymes in the alcohol biosynthesis pathways, the carbon metabolic flux of substrates is generally distributed unfavorably between alcohol biosynthesis and other competing pathways [18–20]. This leads to a much lower yield of the target alcohol from sugars in the actual production process.

Therefore, to improve alcohol production, and especially the yield that can be achieved from cheap substrates, cellular redox homeostasis must be manipulated to avoid a possible limitation of reducing equivalents. In this article, we review recent advances in accelerating the production of alcohols by engineering microbial redox homeostasis, including providing sufficient amounts of needed cofactors, improving the affinity of key enzymes to the available reducing equivalents, manipulating the intracellular electron transport chain, and other approaches for engineering the cellular redox balance.

### Improving the availability of required cofactors to enhance cofactor-dependent alcohol production

Targeted regulation of enzymes or genes involved in the target pathway is often the first step in metabolic engineering of microbes for alcohol production. However, once the enzyme levels are no longer limiting, cofactor availability can become the main bottleneck for cofactor-dependent redox reactions [21]. Nicotinamide adenine dinucleotide (NAD) functions as a cofactor in over 300 oxidation–reduction reactions and regulates various enzymes and genetic processes [21]. The NADH/NAD^+^ cofactor pair also plays a major role in microbial catabolism [22]. Due to their role as co-substrates, the concentration of cofactors, together with other substrates, determines the rate of enzymatic reactions and therefore the flux of the corresponding pathway. Many strategies have thus been developed to improve the availability of cofactors such as NADH and NADPH, and successfully applied to enhance the microbial production of various alcohols.

#### Fine-tuning of genes expression in alcohol biosynthetic pathway to enhance NAD(P)H competitiveness

Usually, there would be more than one enzyme involved in the alcohol synthetic pathway. Thus a proper proportion of these enzymes especially the NAD(P)H-dependent one is of crucial role. Fine-tuning of gene expression through manipulation of mRNA stability [23], modulation of the ribosome binding site (RBS) [24], codon optimization [25] and other approaches [26, 27] can be benefit for the redox balance in alcohol-producing cells.

Fine-tuning of *GRE3* which is strictly NADPH-dependent expression could be more useful to reduce xylitol formation and increase ethanol production from xylose in *Saccharomyces cerevisiae* [28, 29]. Meanwhile, fine-tuned overexpression of xylulokinase in *S. cerevisiae* could lead to improved fermentation of xylose to ethanol [29] and fine-tuning of NADH oxidase could decrease byproduct accumulation in *S. cerevisiae* [30]. Sun et al. engineered a 1,2,4-butanetriol-producing *Escherichia coli* and fine-tuned the expression of *yjhG* and *mdlC*. The relative strain BW-026 increased 1,2,4-butanetriol titer by 71.4% [4]. Recently, Ohtake et al. [31] engineered a high titer butanol-producing *E. coli* strain by fine-tuning of *adhE2* which is NADH-dependent. The authors believed a CoA imbalance problem was solved improving the butanol production. On the other hand, the redox balance was also further achieved as *adhE2* is responsible for two steps consuming NADH in butanol synthetic pathway.

#### Blocking of competing NADH-withdrawing pathways to redirect metabolic flux towards the target alcohols

In many microorganisms, and most production strains, glycolysis is the key upstream pathway in the fermentation process from sugars to alcohols, with pyruvate as the node linking different directions of carbon flow. Concomitantly with the generation of pyruvate, a net two NADH molecules are generated from one glucose molecule [32]. To return this reduced cofactor to its oxidized state, oxidative phosphorylation or anaerobic fermentation is implemented to generate ATP or reduced byproducts, respectively [18]. In *E. coli*, lactate, ethanol, succinate, amino acids, and some other chemicals can be derived from pyruvate [or phosphoenolpyruvate (PEP)], consuming NADH under anaerobic conditions [33]. Hence, a direct approach to provide more NADH for alcohol formation is to block the pathways competing for it.

Lactate can be directly generated from pyruvate and NADH with no additional intermediate reactions, thus making it a very competitive byproduct that needs to be removed. Berríos-Rivera et al. [19] showed that an *ldh* ^−^ genotype increased the synthesis of 1,2-propanediol (1,2-PDO) in *E. coli*, which was considered an NADH-limited system. This work manipulated the NADH/NAD^+^ pool by eliminating the competing lactate pathway, which provided a more reducing environment for alcohol production [19]. Likewise, Zhang et al. inactivated the *aldA* gene encoding ALDH, an enzyme that competes with 1,3-propanediol (1,3-PDO) oxidoreductase for NADH in *Klebsiella pneumoniae*, to produce higher amounts of 1,3-PDO. By this manipulation, the product titer was increased by 33% compared with the control strain, and the yield of 1,3-PDO from glycerol was increased from 0.355 to 0.699 mol/mol, reaching an astonishing 97.1% of the maximal theoretical yield [34]. Similar effects were found in the engineered butanol-producing strains. By deleting the main competing NADH-withdrawing pathway genes in *E. coli*, including *adhE* for ethanol, *ldhA* for lactate, and *frdBC* for succinate, butanol production was significantly improved, leading to a doubling of the titer. After additionally blocking other byproduct pathways, the final butanol titer of the resulting strain increased by 133% [20, 35, 36].

An approach guided by in silico metabolic engineering of *E. coli* for direct production of 1,4-butanediol (1,4-BDO) also led to a strategy of eliminating pathways which compete for reducing power [37, 38]. Similarly, Fu et al. pointed out that although the deletion of *ldh* did not increase the metabolic flux towards the 2,3-butanediol (2,3-BDO) pathway, it increased the NADH/NAD^+^ ratio for further conversion of acetoin to 2,3-BDO, underscoring that NADH availability was the key factor for 2,3-BDO production [39].

#### Increasing the total level of NAD to accelerate alcohols production

The total level of NAD (NAD^+^ and NADH) is strictly controlled in microorganisms through specific regulatory mechanisms [40]. A de novo pathway and a pyridine nucleotide salvage pathway was found in *E. coli* to maintain its total intracellular NADH/NAD^+^ pool [40]. Berríos-Rivera et al. found that the nicotinic acid phosphoribosyltransferase, encoded by the *pncB* gene, can catalyze the formation of a precursor of NAD. Consequently, they overexpressed the *pncB* gene from *Salmonella typhimurium* to increase the total level of NAD. Anaerobic tube experiments showed that the strains overexpressing *pncB* had higher biomass and increased ethanol/acetate ratios [40]. Jawed et al. [41] also performed this *pncB*-overexpressing method in a *Klebsiella* HQ-3 strain and observed increased production and yield of H_2_. Along with H_2_, 2,3-BDO and ethanol titers were improved as well due to the increased availability of NADH [41]. Another study showed enhancement of succinate production by expressing nicotinic acid phosphoribosyltransferase gene *pncB* [42]. Although it is not alcohol related, succinate is a reducing chemical which makes it a valuable reference.

#### Regeneration of NAD(P)H to increase the availability of its reduced form to accelerate alcohol production

In addition to the total NAD(P) pool, the ratio of the reduced to the oxidized form will determine the reaction activity. Reduced cofactors (NADH, NADPH, reduced ferredoxin) are needed to provide electrons for the reduction of precursors to alcohols [43]. Therefore, efficient regeneration of NAD(P)H is crucial for optimal production of alcohols, especially in anaerobic fermentations.

Several enzymatic methods have been developed for the regeneration of NADH [44]. By overexpressing the NAD^+^-dependent formate dehydrogenase (FDH) from *Candida boidinii* in *E. coli*, the maximum yield of NADH was doubled from 2 to 4 mol NADH/mol glucose consumed [21]. Compared with the control strain, the ethanol to acetate (Et/Ac) ratio of the engineered strain containing heterologous FDH increased dramatically, by nearly 30-fold. What makes it even more interesting is the observation that the increased availability of NADH induced the production of ethanol even in the presence of O_2_, and the amount of ethanol was dependent on the amount of added formate [21]. This approach was also demonstrated to be effective for improving the Et/Ac ratio in minimal medium [22]. Similarly, the *fdh* gene was introduced into *Klebsiella oxytoca*. Interestingly, in said case both the oxidative and the reductive metabolism of glycerol was enhanced [45]. Results indicated that the engineered strain OF-1 produced more 1,3-propanediol, ethanol, and lactate than the control strain, as a result of increased NADH availability. The molar yield of 1,3-PDO was 17.3% higher than that of the control strain [45]. Using the same formate/formate dehydrogenase NADH regeneration system, the target pathways of (2S,3S)-2,3-butanediol [46] and butanol [47, 48] were effectively coupled to the NADH driving force, respectively, and the product titers were also improved significantly.

In addition to fine-tuning *fdh1* expression levels, it was demonstrated that the intracellular redox state could be modulated by anaerobically activating the pyruvate dehydrogenase (PDH) complex. The engineered strain showed the highest reported butanol productivity from glucose in *E. coli* (0.26 g/L/h) [35]. It indicated a new approach to improve the availability of NADH.

In spite of NADH, there are strategies reported on NADPH regeneration for alcohols or reduced chemicals production. Verho et al. expressed a discovered *GDP1* gene coding an NADP^+^-dependent d-glyceraldehyde-3-phosphate dehydrogenase for ethanol fermentation in *S. cerevisiae* [49]. The *GDP1*-overexpressed strain produced ethanol with a higher rate and yield than the control strain. Combining with the deletion of *ZWF1* (coding glucose-6-phosphate dehydrogenase for NADPH and CO_2_ generation) for redox balance, the resulting strain produced 11% more ethanol and 69% less xylitol which is the main byproduct in xylose fermentation [49]. Furthermore, glucose dehydrogenases from different microorganisms were also used for NADPH regeneration [50, 51]. Eguchi et al. used a glucose dehydrogenase cloned from *Gluconobacter scleroides* for recycling of cofactor NADPH in vitro [50], while Xu et al. cloned a glucose dehydrogenase gene *gdh* from *Bacillus megaterium* to regenerate NADPH in vitro and in vivo [51]. A recent study also reported an approach to enhancing NADPH supply by overexpressing glucose-6-phosphate dehydrogenase [52]. These examples demonstrated the possibility of engineering the regeneration of NADPH for efficient alcohol production.

In addition to the purely bio-catalytic regeneration of NADH and NADPH, electricity-driven NAD(P)H regeneration and direct electron transfer are rapidly being developed and have been applied experimentally for CO_2_ fixation in the recent 5 years [53–56]. These studies focused on the delivery of electrons from electrodes to the cells to supply reducing power, which in turn can be used for alcohol production [57]. CO_2_ is an oxidizing compound which requires large amounts of energy and reducing power to be fixed into organics. In nature, cyanobacteria and higher plants use NADPH to fix CO_2_ in the Calvin cycle [58, 59]. Li et al. [53] designed an integrated electro-microbial process to convert CO_2_ into formate, which was further turned into NADH by formate dehydrogenase. The generated NADH was used for isobutanol synthesis in *Ralstonia eutropha*. About 846 mg/L isobutanol was produced, indicating the tantalizing possibility of microbial electrosynthesis of alcohols. Torella et al. [55] reported a hybrid microbial water-splitting catalyst system which was similar to natural photosynthesis. In this system, water was electrolyzed by electricity for the supply of reduced cofactors (NADPH) with the help of hydrogenases, and CO_2_ was fixed through the Calvin cycle in an engineered *R. eutropha* strain using the obtained NADPH. Using this system, 216 mg/L isopropanol was synthesized with high selectivity [55].

In the above content, we listed some approaches of improving the availability of needed cofactors for alcohol production and described each approach respectively. However, these approaches are not always separately employed in metabolic engineering for alcohol production. Blocking of competing NADH-withdrawing pathways was usually accompanied by introduction of NADH regeneration systems [35]. Analogously, fine-tuning of gene expression may connect with introduction of NADH regeneration systems in alcohols synthetic pathway [31, 48]. Additionally, the strategy of increasing the total level of NAD can conceivably be combined with the introduction of an NADH regeneration system to exert an even stronger effect [60]. Therefore, in systems metabolic engineering of alcohol production, different kinds of cofactor engineering approaches could be considered and combined.

### Manipulating the affinity of key redox enzymes for NADH or NADPH to improve alcohol production

**Fig. 1:**
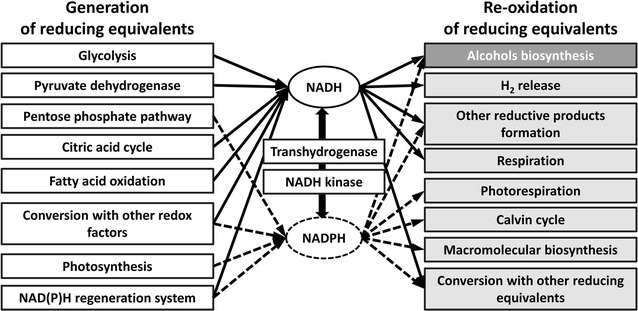
Common NAD(P)H-dependent metabolic pathways in microbes. *Dashed arrow line*: NADPH; *solid arrow line*: NADH

In cells, various redox enzymes prefer different reducing equivalents. NAD(H) and its phosphate form NAD(P)H play major roles in metabolic processes of all living beings [21]. In microorganisms, over 400 redox enzymes have a high affinity to NAD(H) and another 400 ones have a high affinity to NADP(H), they are dependent on NAD(H) and NADP(H), respectively [38, 61]. In addition, some redox enzymes are dependent on ferredoxin, the flavin nucleotides flavin-adenine dinucleotide (FAD) and flavin mononucleotide (FMN), heme, pyrroquinoline quinone (PQQ) or other cofactors [38, 62]. As shown in Fig. 1, NADH and NADPH can be generated from different pathways in microbes. In any case, the electron balance must be satisfied and thus reduced electron carriers, like NADH and NADPH, must be re-oxidized, mostly via the reduction of substrates to alcohols, or the formation of H_2_ and/or other reductive metabolites [43]. Commonly, electrons are transferred between the reduced and oxidized forms of the cofactor, the corresponding redox enzyme and the reactants, forming a redox cycle. However, it is also possible that some of the proteins mediate the exchange of electrons between NADH, NADPH, ferredoxin and other reducing equivalents. Sometimes, the types of reducing equivalents generated from the available substrates are not fit for the redox enzymes that re-oxidize the necessary cofactors [11, 63]. Thus, to meet the redox requirements for alcohol biosynthesis, it is necessary to construct novel redox cycles and therefore to achieve new redox homeostasis. Recently, many attempts have been made to change the affinity of key redox enzymes for different types of reducing equivalents, or to interconvert the reducing equivalents between different types.

#### Switching the redox enzymes’ affinity from one type of reducing equivalent to another to efficiently couple alcohol production to cellular redox homeostasis

As described above, NAD(H) is the most abundant reducing equivalent in most bacteria and yeasts. Consequently, many efforts have been made to change the preferential affinity of redox enzymes from NADPH to NADH. For example, using xylose as a feedstock to produce ethanol in *S. cerevisiae* has attracted much attention, and it was found that the ethanol yield was far below the theoretical maximum because of imbalanced coenzyme utilization [63]. An NADPH-preferring xylose reductase (XR) and a strictly NAD^+^-dependent xylitol dehydrogenase (XDH) caused the cofactor imbalance, leading to a low yield. Consequently, researchers employed structure-guided site-directed mutagenesis to change the coenzyme preference of *Candida tenuis* XR from NADPH in the wild-type enzyme to NADH [63, 64]. The strain harboring the resulting XR double mutant showed a 42% enhanced ethanol yield (0.34 g/g) compared to the reference strain harboring wild-type XR, in anaerobic bioconversions of xylose [63]. Likewise, the NADH preference of *Pichia stipitis* XR could also be altered by site-directed mutagenesis [65]. An engineered XR with the point mutation K270R was combined with the capability of xylose utilization, and the resulting *S. cerevisiae* gave an ethanol yield of 0.39 g/g and a titer of 25.3 g/L, which was 18 and 51% higher than the reference strain, respectively [65].

Generally, NADH is the preferred electron donor for redox enzymes in most organisms, but some are capable of efficiently generating NADPH. An example of this are photosynthetic cyanobacteria [66]. For these microbes, the use of NADPH-dependent enzymes can be beneficial for alcohol production. Lan and Liao introduced the butanol pathway into *Synechococcus elongatus* PCC 7942 by exchanging the NAD^+^-dependent enzymes with NADP^+^-dependent ones, enabling them to consume the NADPH generated through photosynthesis [67]. By coupling the pathway with an ATP-driven step, the cyanobacterial strain successfully produced 29.9 mg/L butanol, increasing butanol production by fourfold [67]. Interestingly, by introducing an additional NADPH-consuming isopropanol synthetic pathway, the photosynthesis of *Synechocystis* sp. PCC 6803 was improved by about 50%, due to the immediate re-oxidation of NADPH that was generated from the photoreaction. At the same time, 226.9 mg/L of isopropanol was produced by this engineered strain [68]. Considering their ethanol-producing potential [57], cyanobacteria may well become the most cost-effective alcohol producing microbial cell factory in the future [69].

Dai et al. [70] introduced a single secondary alcohol dehydrogenase into *C. acetobutylicum* to consume NADPH for isopropanol production which switches ABE fermentation to a higher level IBE fermentation. The clostridial butanol synthesis pathway utilizes both NADH and reduced ferredoxin as sources of reducing power [71]. In order to couple the NADH driving force to the butanol pathway, a *trans*-enoyl-CoA reductase (Ter) was chosen to replace the butyryl-CoA dehydrogenase complex (Bcd-EtfAB), and thus to balance the reducing power in the form of NADH. The resulting strain produced 1.8 g/L of butanol in 24 h compared to only 0.1 g/L generated by an equivalent construct harboring Bcd-EtfAB [48].

Interconverting the reducing equivalents between different types is also a promising strategy to meet the redox requirements for the biosynthesis of target products. Panagiotou et al. demonstrated that the overexpression of an ATP-dependent NADH kinase to convert NADH into NADPH had a positive effect on growth efficiency in *Aspergillus nidulans*. Since aspergilli are major players in industrial biotechnology, it is conceivable that this strategy could enable the development of many new strains capable of generating the important reducing power in the form of NADPH, which is crucial for efficient production of metabolites and enzymes in large-scale fermenters [72]. In some cases, NADPH is needed directly for the production of target chemicals. For this purpose, researchers have genetically engineered an *E. coli* strain to increase the availability of NADPH by replacing the native NAD^+^-dependent glyceraldehyde-3-phosphate dehydrogenase (GAPDH) with an NADP^+^-dependent GAPDH from *C. acetobutylicum*. This resulted in the generation of 2 mol of NADPH, instead of NADH, per mole of glucose consumed [11].

Taking an approach that is different from engineering the affinity for natural cofactors, recently, Zhao et al. created artificial redox systems which depend on nicotinamide flucytosine dinucleotide and showed excellent activity with the NAD-dependent malic enzyme [73]. This opens a new avenue for engineering bioorthogonal redox systems for a wide variety of applications in systems and synthetic biology, which could also be implemented in alcohol production [38].

#### Engineering of key enzymes to improve their affinity for NAD(P)H and decrease the redox requirements for alcohol production

During the production of alcohols, some cofactor-dependent key enzymes are often rate-limiting, which is obviously unfavorable. Ingram et al. found more effective enzymes utilizing NADH in ethanol production. Alcohol dehydrogenase II and pyruvate decarboxylase from *Zymomonas mobilis* were expressed at high levels in *E. coli*, resulting in increased cell growth and the production of ethanol as the principal fermentation product from glucose [18].

In addition to substituting intrinsic enzymes with more efficient ones, direct engineering of target enzymes to improve their affinity for specific cofactors is also a practical way to increase the product titer of various alcohols. Directed evolution which is a method for protein engineering and protein evolution mimicking natural selection has often been performed to engineer the characteristics of target enzymes [74]. Bastian et al. engineered an NADH-dependent IlvC by directed evolution, and coupled it with an engineered *Lactococcus lactis* AdhA in the isobutanol pathway. The *K*_m_ value of this IlvC variant for NADH was dramatically decreased from 1080 to 30 μM, which was even lower than the *K*_m_ of its native substrate NADPH which is 40 μM. At the same time, the engineered AdhA also showed increased affinity for NADH, with a change in *K*_m_ value from 11.7 to 1.7 mM. Strains carrying the two engineered enzymes improved the yield to practically 100% of the theoretical limit under anaerobic conditions using glucose as feedstock [75].

Structure-based rational design is also an important approach used to engineer enzymes. Meng et al. recently engineered the affinity of a d-lactate dehydrogenase for NADH and NADPH. Based on computational design and analysis, the wild-type NADH-dependent d-lactate dehydrogenase from *Lactobacillus delbrueckii* was rationally mutated to increase its affinity for both NADPH and NADH. The mutant enzyme was able to super-efficiently utilize both NADPH and NADH as cofactors [76]. This study is not directly related to alcohol production, yet it may provide useful reference points.

### Engineering the cellular redox environment at a global level to benefit alcohol production

As described above, fermentations for alcohol production are mostly performed under anaerobic conditions. In the presence of sufficient O_2_, most industrial organisms use active respiration to re-oxidize NADH and O_2_ is usually used as the final electron acceptor. Furthermore, under some conditions, O_2_ can lead to the production of free radicals from the electron transport chain, which can cause severe stress to microorganisms [77]. This in turn can indirectly hinder alcohol production. Some strategies have been reported to improve alcohol production by blocking O_2_-mediated NADH oxidation and strengthening the redox balance [78, 79].

#### Manipulating respiratory levels to redirect the electron transport chain towards aerobic formation of alcohols

Under aerobic conditions, cells produce large amounts of ATP through respiration, and grow rapidly, but alcohol production is inhibited due to a lack of NADH. Zhu et al. reported a smart strategy to limit respiratory levels, allowing the formation of reduced chemicals such as ethanol even under fully aerobic conditions. By knocking out the *ubiCA* genes, which encode two critical enzymes for ubiquinone synthesis and therefore respiration in *E. coli*, and by supplementing external coenzyme Q1, the respiratory level was manipulated so that up to 80% of the carbon atoms from glycerol were converted into ethanol [78]. It thus demonstrated that NADH (redox) partitioning between energy generation in the electron transport chain (respiration) and the use of NADH for reduction of metabolic intermediates could be precisely controlled.

In addition to genetically manipulating respiratory levels to redirect the electron transport chain, electron carriers based on artificial chemicals have also been used to direct electron flow. Stoichiometric network analysis revealed that NAD(P)H that was lost from the fermentation in the form of H_2_ limited the yield of butanol, and led to the accumulation of acetone. By using methyl viologen as an electron carrier to divert the electron flow away from H_2_ production, the NAD(P)H supply was reinforced, which increased butanol yields by 37.8%, along with strongly diminished acetone production [79].

#### Introduction of glutathione to enhance the thiol redox balance and accelerate alcohol biosynthesis

In addition to its direct participation in NAD(P)H-dependent reactions, these cofactors also play a prominent role in the physiological functions linked to microbial growth and metabolism. As the storage molecules of reducing power, NADH and NADPH provide most of the electrons that reverse O_2_-dependent thiol oxidation, constituting the thiol redox system, together with the glutathione (GSH, l-γ-glutamyl-l-cysteinylglycine) and thioredoxin pathways [12], which control intracellular redox homeostasis. Correspondingly, the microbial thiol redox system, including GSH, is presumed to affect the NADH and NADPH availability and therefore control the flux of NAD(P)H-dependent pathways.

GSH is the most abundant non-protein thiol, and is widely distributed in living organisms [80]. It plays important roles in many physiological and metabolic processes, including thiol redox homeostasis, protein stabilization, antioxidation, stresses tolerance and provision of electrons to reductive enzymes via NADPH [81–83]. The biosynthesis of GSH involves two consecutive enzymatic reactions, catalyzed either by the two separate enzymes γ-glutamylcysteine synthetase (γ-GCS, encoded by *gshA*) and GSH synthetase (GS, encoded by *gshB*), or by a bifunctional γ-glutamate-cysteine ligase/GSH synthetase (GshF). By over-expressing the *gshAB* genes from *E. coli*, GSH biosynthetic capability was introduced into *C. acetobutylicum* DSM 1731, and the resulting strain produced 14.8 g/L butanol, which was 37% higher than its wild-type parent. The engineered strain also exhibited improved tolerance to aeration and butanol [84]. This strategy was also applied in the butanol-producing strain *C. acetobutylicum* ATCC 824. By expressing the *gshAB* genes from *E. coli* in the *adc* locus, butanol production in the engineered strain 824*adc*::*gsh* was increased by 59%, reaching 8.3 g/L [85].

#### Engineering the redox-sensitive transcription factor Rex to control NADH/NAD^+^ homeostasis in order to manipulate alcohol biosynthesis

Anaerobic microbes, such as *C. acetobutylicum*, have evolved a number of strategies to cope with the oxidative stress from reactive oxygen species and molecular O_2_. In addition to the protection provided by GSH, it was found that reducing equivalents directly participate in the defense against oxidative stress in *Clostridium* by reducing O_2_ and oxygen free radicals, which favorably shifts the cellular redox balance [14, 86]. Interestingly, the redox-sensing transcriptional repressor Rex has recently been found to play a role in the solventogenic shift of *C. acetobutylicum* [87]. Rex is composed of two domains, an N-terminal winged-helix DNA-binding domain and a C-terminal Rossmann-like domain involved in NADH binding and subunit dimerization. The DNA-binding activity of Rex protein is modulated by the ratio of NADH to NAD^+^ [88, 89]. By systematically investigating the Rex regulons in 11 diverse clostridial species, Zhang et al. [14] suggested that Rex plays an important role in maintaining NADH/NAD^+^ homeostasis. This indicates a possible method to improve NADH-dependent alcohol production in clostridia.

## Conclusions and perspectives

The main recent advances on engineering redox homeostasis to accelerate alcohol biosynthesis, from the viewpoints of cofactors availability, enzyme affinity to cofactors and global redox regulation, have been summarized in this article. A number of approaches, as reviewed here, demonstrate the power of redox homeostasis to improve alcohol production. The strategy of improving the availability of the required cofactors can increase both the titers and yields of the desired alcohols to different extents. Although the productivity data are usually not indicated, an increased titer mostly also indicate increased productivity [34, 35, 46]. Manipulating the affinity of key redox enzymes for NADH or NADPH is an effective strategy to meet the specific cofactor requirements for alcohol biosynthesis and yield improving [63, 65, 75]. Globally engineered cellular redox state benefited the microbes’ tolerance to serious stresses, and therefore indirectly benefited the production of alcohols [78, 84, 87]. By employing these approaches, the alcohol production improvements were truly profound in certain cases, and are reflected by the final titers, yields and productivities (Table 1).

**Table 1:** Strategies for engineering redox homeostasis and its effects on alcohols production

Strategy	Specific approach	Target product	Main effects			Ref.
			Titer	Yield	Productivity	
Improving the availability of cofactors						
Fine-tuning of NAD(P)H-dependent gene	Fine-tuining of *yjhG* and *mdlC*	1,2,4-Butanetriol	Increased by 71.4%	NR	NR	[4]
	Fine-tuning of *adhE2*	Butanol	Increased from 15 to 18.3 g/L	NR	NR	[31]
Blocking NADH-competing pathways	Knock out *ldh*	1,2-Propanediol	Increased from 1.08 to 1.30 g/L anaerobically, from 1.10 to 1.40 g/L microaerobically	Increased by 43% anaerobically, by 67% microaerobically	NR	[19]
	Knock out *aldA*	1,3-Propanediol	Increased from 698.6 to 927.6 mM	Increased from 0.355 to 0.699 mol/mol	Increased by 33%	[34]
	Knock out *adh*, *ldh* and *frd*	Butanol	Increased from 141 to 274 mg/L	NR	NR	[20]
	Knock out *mdh*	1,4-Butanediol	Increased from ~3 to ~8 mM	NR	NR	[37]
Increasing total NAD level	Overexpress *pncB*	Ethanol	Increased from 11.50 to 28.58 mM	NR	NR	[40]
Introducing NAD(P)H regeneration systems	Overexpress *fdh1*	Ethanol	Increased from ~15 to ~175 mM	NR	NR	[21]
	Overexpress *fdh1*	Ethanol	Increased from 52.20 to 117.77 mM	Increased from 0.72 to 1.33 mol/mol	NR	[22]
	Overexpress *fdh*	1,3-Propanediol	NR	Increased by 17.3%	NR	[45]
	Overexpress *fdh*	2,3-Butanediol	Increased from 16.1 to 17.8 g/L	Increased from 82.5 to 91.8%	Increased by 33%	[46]
	Activate pyruvate dehydrogenase, fine-tune express *fdh1*	Butanol	Increased from 5.02 to 6.8 g/L	NR	Increased by 136%	[35]
	Overexpress *GDP1*	Ethanol	Increased from 90 to 100 mM	Increased from 18 to 41%	NR	[49]
	Electrically regenerate NADH	Isobutanol	Produced 846 mg/L	NR	NR	[53]
	Electrically regenerate NADPH	Isopropanol	Produced 216 mg/L	NR	NR	[55]
Manipulating affinity of redox enzymes for NAD(P)H						
Switching the affinity from one type to another	Mutate XR (NADPH to NADH)	Ethanol	NR	Increased from 0.24 to 0.34 g/g	NR	[63]
	Mutate XR (NADPH to NADH)	Ethanol	Increased from 16.7 to 25.3 g/L	Increased from 0.33 to 0.38 g/g	NR	[65]
	Introduce NADPH-preferring enzymes in *Synechococcus*	Butanol	Increased from 6.4 to 29.9 mg/L	NR	NR	[67]
	Replace *bcd*-*etfAB* with *ter*	Butanol	Increased from 0.1 to 1.8 g/L	NR	NR	[48]
Improving affinity for NAD(P)H	Introduce alcohol dehydrogenase II and pyruvate decarboxylase genes from *Z. mobilis*	Ethanol	Increased from 18 to 750 mM	NR	NR	[18]
	Increase affinities of IlvC and AdhA for NADH	Isobutanol	Increased from 1 to 13.4 g/L	Increased from 53 to 100% of the theoretical yield	Increased by 38–88%	[75]
Globally engineering cellular redox balance						
Manipulating respiratory levels	Knock out *ubiCA* and supply coenzyme Q1	Ethanol	NR	Increased from 0.48 to 0.80 mol/mol aerobically	NR	[78]
Introducing glutathione	Overexpress *gshAB*	Butanol	Increased from 10.8 to 14.8 g/L	NR	NR	[84]
Engineering redox-sensitive transcription factor Rex	Inactivate *rex*	Ethanol, Butanol	Increased from ~20 to ~120 mM and increased from 60 to 120 mM, respectively	NR	NR	[87]

*NR* not reported

Redox homeostasis engineering may play an important role in developing alcohol-producing microbial cell factories, yet it is not omnipotent. Firstly, it is hard to quantify the exact impact of cofactor manipulation on reducing equivalents as some unknown formats of reducing equivalents exist not only NAD(P)H, FADH_2_, etc. [90, 91]. Consequently, some strategies could be useless or bring burden to the cells, and sometimes may even be harmful to the cell hosts [92]. Secondly, the cellular redox state is dynamically changed and cannot be monitored in real time, which makes it difficult to completely understand the whole process of alcohol production. Thirdly, there are other redox relevant enzymes except for alcohol synthetic pathway enzymes. These enzymes may have physiological function shifting the cell to another metabolic pattern after the above approaches were adopted [93].

Although rapidly advancing, the tools and methods of systems metabolic engineering still await more exciting developments for controlling the metabolic fluxes and energy/redox requirements in the context of maximizing product titer, yield and productivity. Since traditional cofactor engineering might not be sufficient to meet the demand for higher titer, yield and productivity of target products, future work will have to use systems and synthetic biology approaches in order to further understand the redox systems of typical industrially relevant bacteria. In addition, the product yield is always limited by the provided substrate (including co-substrate) due to the stoichiometry of available electrons from a substrate [16]. Engineering of redox homeostasis made it possible to close to the maximal theoretical yield, but it was hardly to obtain a yield beyond the limits from substrate. Reports on other target chemicals have also provided certain reference points for future engineering of redox homeostasis. Feedstocks which are more reduced than glucose may be suitable for the production of alcohols such as glycerol [5] and sorbitol [22], but also fatty acids [17]. Additionally, extracellular redox potential (ORP) was validated as an effective parameter that controls the anaerobic microbial production of 1,3-propanediol [94] and butanol [95]. In the future, improving the metabolic flux towards target products by controlling extracellular ORP could be employed in some reactions which are difficult to conduct, especially ones that need very low redox potentials [96, 97].

## Abbreviations

NADH: reduced nicotinamide adenine dinucleotide
NAD^+^: oxidized nicotinamide adenine dinucleotide
NADPH: reduced nicotinamide adenine dinucleotide phosphate
NADP^+^: oxidized nicotinamide adenine dinucleotide phosphate
RBS: ribosome binding site
PEP: phosphoenolpyruvate
1,2-PDO: 1,2-propanediol
1,3-PDO: 1,3-propanediol
1,4-BDO: 1,4-butanediol
2,3-BDO: 2,3-butanediol
FDH: formate dehydrogenase
Et/Ac: ethanol to acetate
PDH: pyruvate dehydrogenase
FAD: flavin adenine dinucleotide
FMN: flavin mononucleotide
PQQ: pyrroquinoline quinone
XR: xylose reductase
XDH: xylitol dehydrogenase
GAPDH: glyceraldehyde-3-phosphate dehydrogenase
GSH: glutathione
γ-GCS: γ-glutamylcysteine synthetase
GS: GSH synthetase
ORP: redox potential
